# Guide to the Examination of Urine

**Published:** 1879-09

**Authors:** 


### BIBLIOGRAPHICAL.

Guide to the Examination of Urine, with Special Reference to the Diseases of the Urinary Apparatus. By K. B. Hoffman, Prof. at the University of Graz, and R. Ultzman Docent at the University of Vienna. From the Second Edition, Translated and Edited by F. Forcheimer, M.D., Prof. of Med. Chem. at the Med. College of Ohio; with illustrations. Peter G. Thompson, publisher, Cin. Cloth, 1.50; Leather, 2.00. 1879.

This manual, from its being universally adopted by the high-schools of Germany, bears with it the prestige of standard authority. It is a neat volume of about two hundred pages, and presents all the information requisite to a proper and thorough examination of the urine. It is a handy book, and no practicing physician should be without it.

